# A Preliminary Evaluation of Lyophilized Gelatin Sponges, Enhanced with Platelet-Rich Plasma, Hydroxyapatite and Chitin Whiskers for Bone Regeneration

**DOI:** 10.3390/cells2020244

**Published:** 2013-04-26

**Authors:** Isaac A. Rodriguez, Scott A. Sell, Jennifer M. McCool, Gunjan Saxena, Andrew J. Spence, Gary L. Bowlin

**Affiliations:** 1 Department of Biomedical Engineering, Virginia Commonwealth University, 401 West Main St, P.O. Box 843067, Richmond, VA 23284, USA; E-Mails: rodriguezia@vcu.edu (I.A.R.); mccooljm2@vcu.edu (J.M.M.); saxenag@vcu.edu (G.S.); glbowlin@vcu.edu (G.L.B.); 2 Department of Biomedical Engineering, Parks College of Engineering, Aviation, and Technology, Saint Louis University, 3507 Lindell Blvd, St. Louis, MO 63103, USA; E-Mail: ssell@slu.edu; 3 Department of Biology, Virginia Commonwealth University, 1000 West Cary St, Room 126, Richmond, VA 23284, USA; E-Mail: spenceaj@vcu.edu

**Keywords:** tissue engineering, bone graft, gelatin, chitin, hydroxyapatite, platelet-rich plasma, scaffold

## Abstract

The purpose of this study was to perform a number of preliminary *in vitro* evaluations on an array of modified gelatin gel sponge scaffolds for use in a bone graft application. The gelatin gels were modified through the addition of a number of components which each possess unique properties conducive to the creation and regeneration of bone: a preparation rich in growth factors (PRGF, a bioactive, lyophilized form of platelet-rich plasma), hydroxyapatite, and chitin whiskers. Platelet-rich plasma therapy is an emerging practice that has proven effective in a number of clinical applications, including enhancing bone repair through improved deposition of new bony matrix and angiogenesis. As such, the inclusion of PRGF in our gelatin scaffolds was intended to significantly enhance scaffold bioactivity, while the addition of hydroxyapatite and chitin whiskers were anticipated to increase scaffold strength. Additionally, the gelatin sponges, which readily dissolve in aqueous solutions, were subjected to 1-Ethyl-3-[3-dimethylaminopropyl]carbodiimide hydrochloride (EDC) cross-linking, either during or post-gelation, to control their rate of degradation. Scaffolds were evaluated *in vitro* with respect to compressive strength, mass loss/degradation, protein release, and cellular interaction, with results demonstrating the potential of the gelatin gel sponge scaffold for use in the regeneration of bone.

## 1. Introduction

Healthy bone has the unique ability to spontaneously regenerate. However, if the diseased or damaged area exceeds a certain size, bone grafting is needed to regenerate the tissue [1]. Common practices to supplement bone regeneration in larger defects include bone graft biomaterials such as autograft (patient bone), allograft (human cadaver bone), xenograft (animal bone), and synthetic biomaterials (*i.e.*, ceramics, cements, glasses, metals, polymers, and composites) [2,3,4,5,6]. Since each of the above mentioned grafts have their own distinct disadvantages, synthetic biomaterial scaffolds that are biocompatible, biodegradable, porous, bioactive, and mechanically stable have been the focus of research as alternative bone substitutes. 

Collagen and hydroxyapatite (HA) are popular materials when investigating bone scaffolds since their composites mimic the extracellular matrix (ECM) of natural bone [7,8,9,10,11]. More recently, gelatin (denatured collagen) has been used as a replacement for collagen since it is less expensive, easier to obtain, and contains similar functional groups which enhance cellular response [12]. Freeze-dried gelatin sponges have many advantages because they can be designed to fit any size defect/injury site, have the ability to swell and fill a void space, degrade controllably in a range of rates (due to various cross-linking methods) to ensure drug release and mechanical stability, and can be easily modified by incorporating various osteoconductive/osteoinductive materials (*i.e.*, minerals, growth factors, proteins, *etc.*). The addition of bioactive inorganic HA to freeze-dried gelatin sponges creates a bone-like ECM scaffold which allows a more controlled drug delivery system and increases cellular attachment, proliferation, alkaline phosphatase activity, and osteocalcin production [13,14,15]. HA also has the ability to bind to a variety of molecules, including proteins. As a result, scaffolds incorporated with HA provide a more favorable environment through increased adsorption of serum adhesion protein such as fibronectin and vitronectin [16]. Enhanced cellular responses have also been observed with the addition of other minerals (β-tricalcium phosphate, Dicalcium Phosphate Dihydrate), polymers (gellan, poly-lactide-co-glycolide) and proteins (bone morphogenetic protein, Wnt1 inducible signaling pathway protein) in combination with HA [17,18,19].

Other additions such as chitin whiskers (CW, *i.e.*, chitin nanocrystals) and platelet-rich plasma (PRP) have been used to increase the mechanical integrity, bioactivity, and osteogenic potential of scaffolds. In recent years, experiments studying CW have increased due to its availability, nontoxicity, and ability to mechanically reinforce polymer nanocomposites and enhance cell proliferation [20,21,22,23,24,25]. Results of numerous studies have demonstrated the versatility and effectiveness of PRP within wound healing, skin engineering, ligament/tendon engineering, cartilage repair, bone regeneration, and more [26,27,28,29,30,31,32]. Particularly with bone regeneration, the addition of PRP has been found to increase bone density/mineralization, vascularization, and osteogenesis [30,33,34,35,36]. Preparation rich in growth factors (PRGF, a bioactive lyophilized version of PRP) contains high concentrations of growth factors such as platelet-derived growth factor (PDGF), transforming growth factor-β (TGF-β), insulin-like growth factor-1 (IGF-1), vascular endothelial growth factor (VEGF), epidermal growth factor (EGF), fibroblast growth factor (FGF), and more [37,38,39]. PRGF also contains cell adhesive proteins such as fibronectin and vitronectin. In relation to bone remodeling, these growth factors and proteins elicit a favorable cellular response which supports the incorporation of PRGF within scaffolds intended for bone tissue engineering [40,41,42,43]. The bone remodeling functions for some of these molecules are summarized in Table 1. The incorporation of PRGF within gelatin sponges, the general combination of CW and PRGF, and the combination of PRGF with HA and/or CW in gelatin sponges are all areas that have yet to be explored.

**Table 1:** Role of PRGF components in bone remodeling [37,38,39,40,41,42,43].

	Function
PDGF	Mesenchymal stem cell and progenitor cell recruitment, proliferation, migration and differentiation into osteoblasts
TGF-β	Mesenchymal stem cells differentiation, increased production of collagen and mineral matrix
IGF-1	Osteoblast proliferation and differentiation
VEGF	Angiogenesis, Endochondral ossification
Fibronectin, Vitronectin	Enhance formation of focal adhesions by osteoblasts, osteoblast migration

The present study aimed to evaluate the release kinetics, degradation, mechanical properties, and cellular responses of multiple combinations of composite freeze-dried gelatin sponges. PRGF, CW, and/or HA were incorporated into the gelatin sponges and cross-linked during gelation or after lyophilization to increase the scaffolds’ overall compatibility as a bone tissue engineering substitute.

## 2. Results and Discussion

### 2.1. Mass Loss

**Figure 1:**
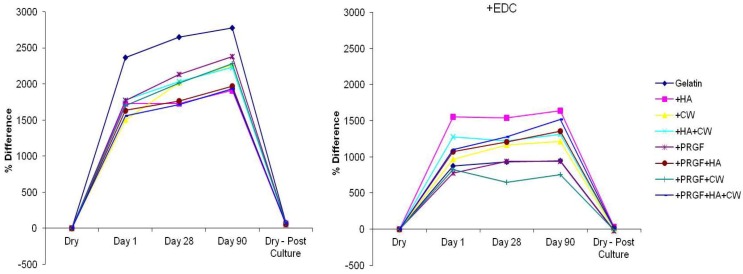
Percent difference in mass.

Figure 1 below shows the percent difference in mass between original dry scaffold weight, days 1, 28, 90, and dry post-culture scaffold weight. When comparing the two methods of cross-linking, scaffolds cross-linked post-fabrication absorbed more media and gained more mass (between 1,500%–2,800%) than scaffolds that were cross-linked during gelation (+EDC, between 500%–1,600%). Upon comparing post cross-linked scaffolds at days 1, 28, and 90, the gelatin scaffolds showed significantly higher (*p* < 0.05) mass increase than any other scaffold type. Also, CW and all scaffolds containing PRGF showed statistically significant (*p* < 0.05) increases in mass at day 90 when compared to day 1, suggesting that these scaffolds continuously absorbed media throughout the culture period. For +EDC scaffolds, most scaffold types reached their maximum weight after day 1 and did not increase in mass over 90 days incubation. Only CW and PRGF+HA+CW scaffolds showed significant (*p* < 0.05) mass increases. On days 1 and 28, HA scaffolds had significantly higher (*p* < 0.05) percent differences in mass than all other scaffolds except for HA+CW and PRGF+HA+CW scaffolds, respectively. After 90 days, the percent difference in mass of HA scaffolds was only significantly higher than gelatin, PRGF, and PRGF+CW scaffolds. Overall, the post cross-linked scaffolds swelled and absorbed more media than the +EDC scaffolds. 

Table 2 below compares the original dry pre-culture mass (Pre) to the dry, post 90 day culture mass (Post) of each scaffold type for both cross-linking methods. All scaffolds that were cross-linked post-fabrication significantly increased in dry mass after culture. For +EDC scaffolds, all scaffolds containing HA showed significant increases in mass while PRGF scaffolds significantly decreased in dry mass. The reason for this increase in mass following a 90 day culture period is not completely understood, especially since each scaffold demonstrated protein release throughout its culture duration that would be indicative of an overall mass loss. It is hypothesized that the gelatin gels absorbed FBS-resident proteins present in the complete media in which they were incubated, which, upon subsequent drying and removal of any liquid prior to the post-culture massing remained trapped within the scaffold’s structure. The swelling and media absorption seen when dry scaffolds were introduced into culture media demonstrated that the gelatin scaffolds had a tremendous hygroscopic potential, thereby making it feasible that large amounts of FBS-resident proteins could be introduced and retained within the scaffold’s structure. Since the post cross-linked scaffolds absorbed more media than the +EDC scaffolds, the increase in mass for these scaffolds could be attributed to the higher absorption of media resulting in increased resident protein integration with the scaffolds. The post dry mass increase observed in +EDC scaffolds containing HA may be attributed to increased resident protein integration as well, considering that HA is a bioactive nanofiller with the capability of increasing binding sites for proteins, minerals, and cells.

**Table 2:** Mass (mg) of dry scaffolds pre/post-incubation. * denotes post-culture mass is statistically different (*p* < 0.05) than pre-culture mass.

			+EDC	
	Pre	Post	Pre	Post
Gelatin	7.97 ± 0.57	14.27 ± 1.51 *	7.53 ± 1.40	6.20 ± 1.71
+HA	12.17 ± 0.61	19.10 ± 0.78 *	8.17 ± 0.67	11.00 ± 1.31 *
+CW	10.67 ± 0.98	17.13 ± 9.29 *	11.23 ± 0.95	10.63 ± 1.45
+HA+CW	11.20 ± 0.70	19.13 ± 8.39 *	7.23 ± 0.29	7.93 ± 0.95
+PRGF	10.83 ± 0.99	18.53 ± 1.60 *	9.40 ± 0.53	7.13 ± 0.81 *
+PRGF+HA	11. 07 ± 0.45	17.43 ± 0.47 *	8.13 ± 0.76	9.47 ± 0.21 *
+PRGF+CW	9.63 ± 1.01	16.10 ± 0.27 *	8.33 ± 0.50	7.73 ± 0.31
+PRGF+HA+CW	11.33 ± 0.65	18.23 ± 0.40 *	8.10 ± 0.26	9.47 ± 0.25 *

### 2.2. Protein Release

**Figure 2:**
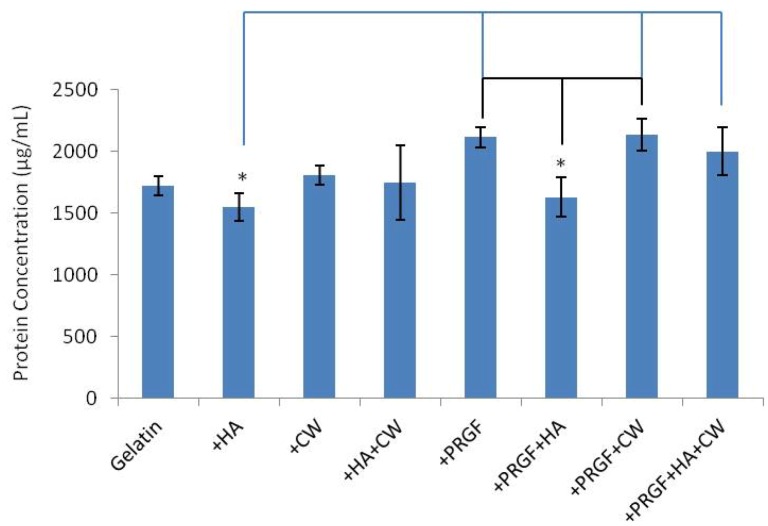
Original protein content of scaffolds. * denotes statistical differences (*p* < 0.05). The black and blue lines show statistical difference of +PRGF+HA and +HA, respectively.

Original total protein content of all scaffolds was between 1,500 and 2,100 µg/mL. The +HA and +PRGF+HA scaffolds contained lower amounts of original protein when compared to other +PRGF scaffolds while all other scaffolds were not statistically different (*p* < 0.05) from each other (Figure 2).

**Figure 3:**
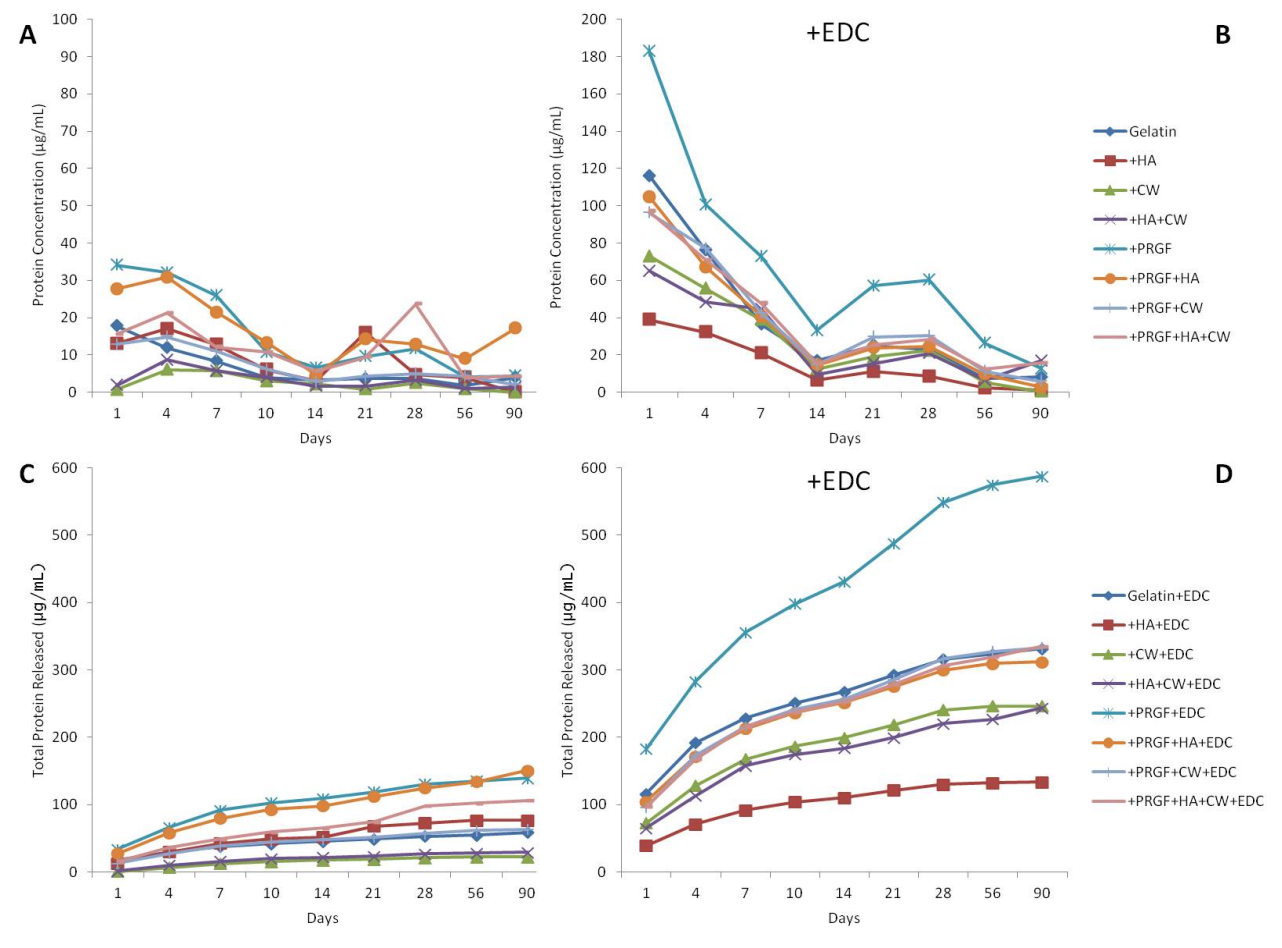
(**A** and **B**) Protein release of scaffolds per time point. (**C** and **D**) cumulative protein release over 90 days.

The protein release kinetics of different cross-linking methods and scaffold compositions are compared over a 90 day incubation period and shown in Figure 3A,B. All +EDC scaffolds released higher amounts of protein after 1 day incubation than their post-gelation cross-linked counterparts. The protein released from +EDC scaffolds ranged between 40 and 185 µg/mL while the post-gelation cross-linked scaffolds only released a maximum of 35 µg/mL. All +EDC scaffolds followed the same trend of protein release; a high release on day 1, a steady decline throughout 14 days, a jump at day 21, then declined to little/no release after 90 days. Throughout 56 days, the +EDC gelatin and +HA scaffolds showed consistently higher and lower release, respectively. Post-gelation cross-linked scaffolds had more variety in trends, however, the overall protein release remained low in comparison to the +EDC scaffolds. Graphs C and D in Figure 3 show the cumulative protein released from scaffolds over 90 days. These graphs show the same trends as graphs A and B; however, it provides a better perspective on overall protein release and scaffold degradation. 

Total protein released from each scaffold after 90 days (Figure 3C,D) was compared to the original protein content of the scaffolds (Figure 2) to compute a percentage of total protein released for each scaffold type after 90 days incubation. Table 3 lists these percentages in descending order. The overall trend was that scaffolds cross-linked during gelation (+EDC) released more protein over 90 days than post-gelation cross-linked scaffolds. These results indicate that the method of cross-linking has a significant impact on the degradation rate of the scaffolds. In this study, where EDC concentrations were identical between the two cross-linking methods (pre or post-gelation) and the only difference was when the cross-linker was applied, it was apparent that the pre-gelation +EDC group were faster degrading than the post-gelation group. While a quick rate of degradation may not be ideal for a bone graft scaffolding material, it may be appropriate in an orthopedic drug delivery scenario where a more rapid, yet still controlled release of protein may be ideal. It should be noted that in this study, for the sake of simplicity, only a single concentration of EDC was utilized. The ability to increase or decrease the concentration of the EDC cross-linker allows users to truly tailor the rate of scaffold degradation by effectively increasing or decreasing the degree to which the scaffold is cross-linked. Future studies will evaluate the chemical interactions and altering EDC concentrations, both pre and post-gelation, to truly optimize the rate of gelatin sponge degradation for a bone graft application. An additional follow up to this preliminary release study will be to conduct an animal implantation study and assess degradation and remodeling of gelatin sponges *in vivo*. While we acknowledge that the *in vitro* behavior may be substantially different from the *in vivo* behavior, this preliminary work was used to narrow the number of materials that we would need to implant in an animal model. By assessing *in vitro* first, we can determine which materials were most effective in vitro and which might be effective *in vivo*.

**Table 3:** Percent total protein released after 90 days.

	% Released
+PRGF+EDC	27.79
Gelatin+EDC	19.30
+PRGF+HA+EDC	19.18
+PRGF+HA+CW+EDC	16.75
+PRGF+CW+EDC	15.62
+HA+CW+EDC	13.94
+CW+EDC	13.66
+PRGF+HA	9.29
+HA+EDC	8.61
+PRGF	6.59
+PRGF+HA+CW	5.32
+HA	4.95
Gelatin	3.43
+PRGF+CW	2.98
+HA+CW	1.68
+CW	1.23

### 2.3. Cell Attachment, Migration and Matrix Production

#### 2.3.1. Scanning Electron Microscopy

**Figure 4:**
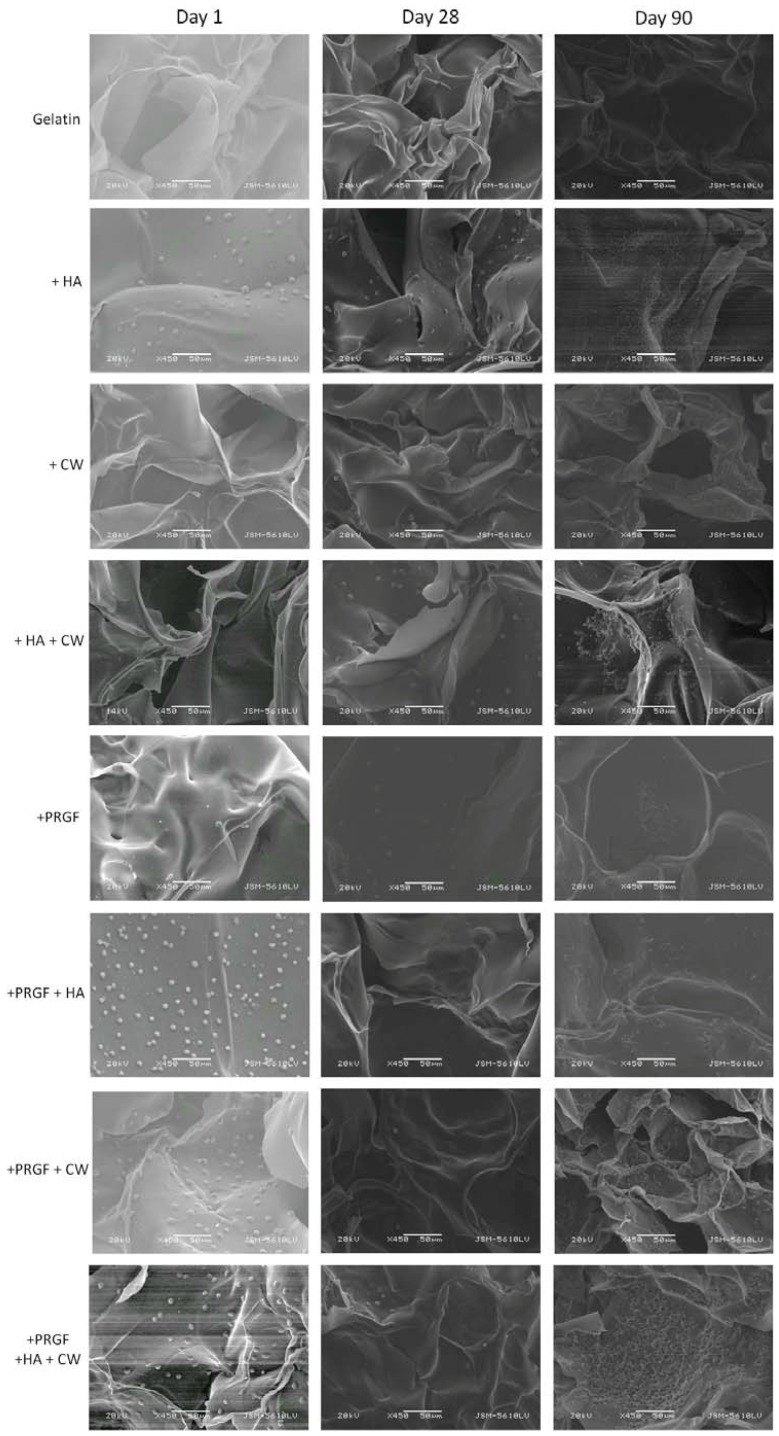
SEM of post cross-linked scaffolds seeded with MG-63 cells for 1, 28, and 90 days. Magnification at 450× and scale bars at 50 µm.

**Figure 5:**
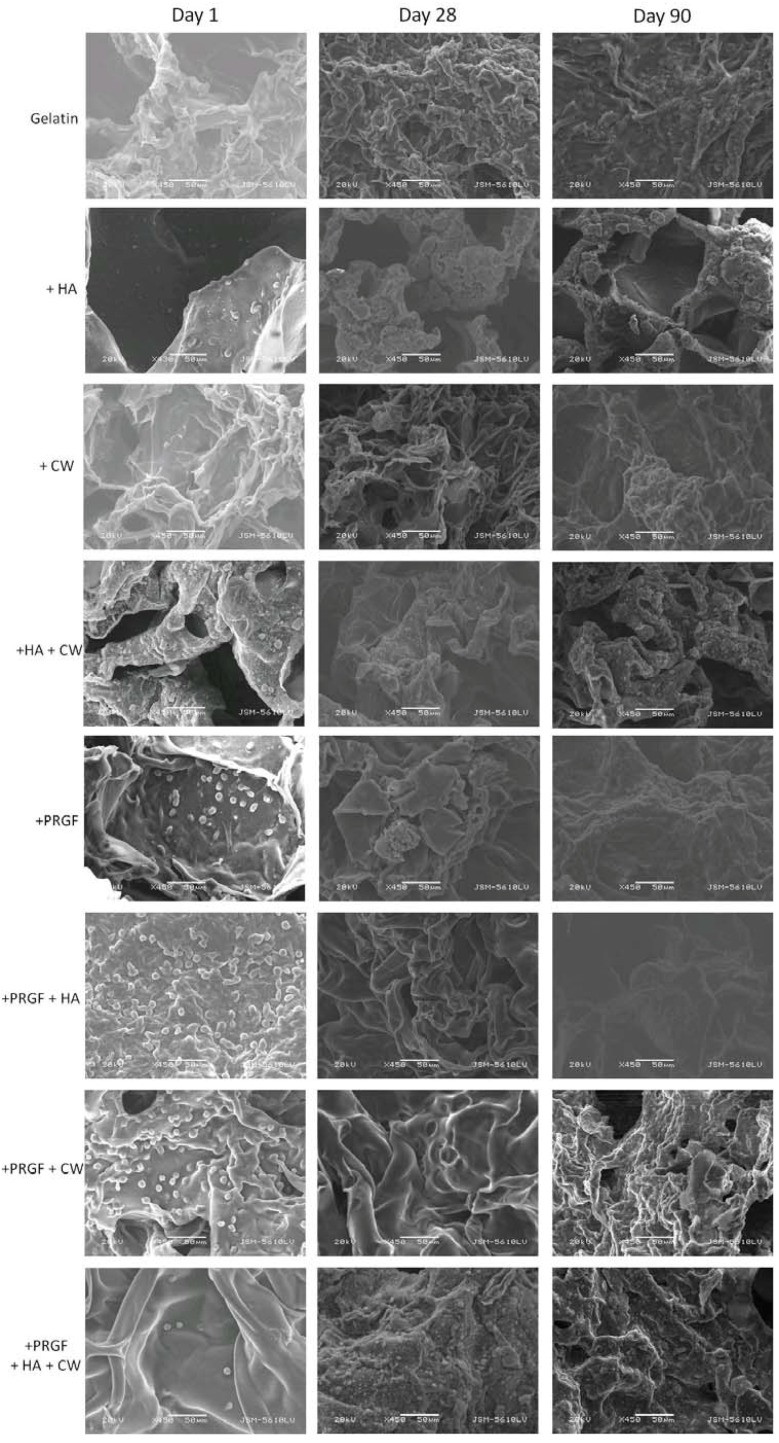
SEM of +EDC cross-linked scaffolds seeded with MG-63 cells for 1, 28, and 90 days. Magnification at 450× and scale bars at 50 µm.

Scaffold characterization, cell attachment, and matrix production was first analyzed via scanning electron microscopy (SEM, Figure 4, Figure 5). Visually, all the acellular scaffolds appeared similar in structure: a flaky surface with large pores. Differences were noticed when observing MG-63 cell attachment after day 1. For post cross-linked scaffolds, the addition of HA and any scaffold containing PRGF appeared to have more cells attached (small dots in image). The same trend was observed for +EDC scaffolds with the exception of +PRGF+HA+CW. As previously mentioned, PRGF contains cell adhesive proteins such as fibronectin and vitronectin. In addition, HA has the ability to adsorb these and other cell adhesion proteins either from the serum once incubated or from the PRGF pre-scaffold fabrication. The presence (in PRGF and serum) and/or adsorption (of HA) of these cell adhesive proteins allow the scaffolds containing HA and/or PRGF to increase in bioactivity and ultimately attach more cells, compared to the gelatin controls. After 28 days, little to no matrix was produced by the cells on the post cross-linked scaffolds, however, the +HA, +HA+CW, +PRGF, and +PRGF+HA+CW +EDC scaffolds showed mineral matrix production by the cells. After 90 days, cells on the post cross-linked scaffolds appeared to produce a more collagen bundle-like morphology matrix rather than a bone-like mineral substance. Cells on +EDC scaffolds for 90 days appeared to have produced more mineral matrix on the surface when compared to post cross-linked scaffolds. By visual inspection of the SEM images, cells produced desired bone-like mineral formation on the +HA, +HA+CW, +PRGF, and +PRGF+HA+CW +EDC scaffolds suggesting these scaffolds to be conducive to bone formation.

#### 2.3.2. Alizarin Red S Staining

**Figure 6:**
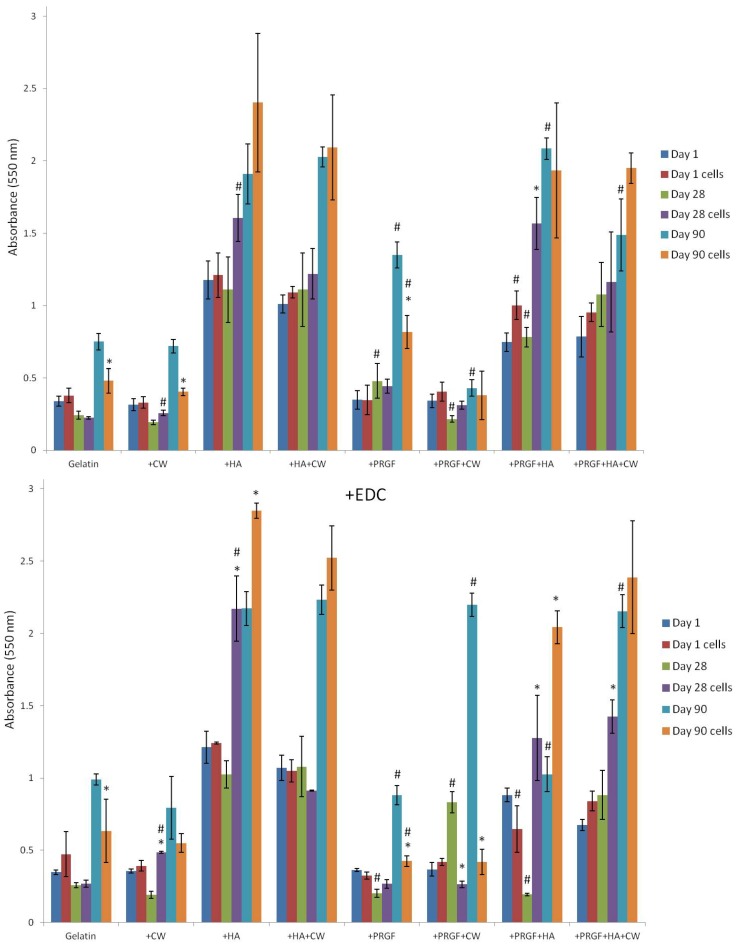
Alizarin Red S staining of acellular and cellular scaffolds over 90 days incubation. * denotes a statistical difference (*p* < 0.05) between cells and no cells for a specific day and scaffold composition. # denotes a statistical difference (*p* < 0.05) when comparing cross-linking methods of each condition.

Absorbance was measured to compare the relative mineral content of the cellular and acellular composite gelatin sponges incubated for 90 days (Figure 6). Each scaffold composition was compared to itself at each day to determine if the addition of MG-63 cells significantly increased or decreased (*p* < 0.05) overall scaffold mineral content. Scaffolds were only compared to themselves and not to other compositions since the initial amount of mineral per scaffold varied (*i.e.*, all scaffolds containing +HA had higher absorbance values than scaffolds without +HA). For post-gelation cross-linked scaffolds, gelatin, +CW, and +PRGF showed a significant decrease (*p* < 0.05) in absorbance after 90 days with cells when compared to 90 days without cells. The only post-gelation cross-linked scaffold that had a significant increase (*p* < 0.05) in mineral content as a result of cells was +PRGF +HA at day 28. For +EDC scaffolds, the addition of cells resulted in significant decreases (*p* < 0.05) in mineral content in gelatin (day 90), +PRGF (day 90), and +PRGF +CW (day 28 and 90) scaffolds. However, there were more +EDC scaffolds that recorded higher absorbance values as a result of the cells producing mineral matrix: +CW (day 28), +HA (day 28 and 90), +PRGF +HA (day 28 and 90), and +PRGF +CW +HA (day 28). 

While the ARS results were not consistent for a specific group, the fact that a number of groups exhibited statistically significant increases in mineral matrix production was seen as a positive result indicative of the potential *in vitro* formation of new bone. The statistically significant decreases between day 90 acellular scaffolds and day 90 cellularized scaffolds could be attributed to the previously mentioned notion of acellular scaffolds continuously absorbing media (and in conjunction, resident proteins and calcium ions within the media) over 90 days. Alternatively, cells seeded on scaffolds degrade the constructs as the cells migrate, proliferate, and produce matrix. The ARS stain may have a higher affinity to these adherent resident proteins and calcium than a partially degraded scaffold covered with cells, resulting in a higher ARS absorbance for acellular scaffolds. Overall, the very nature of the ARS procedure (multiple staining/de-staining and washing steps) makes it difficult to obtain truly accurate quantifications of mineral production. In addition, the porous nature of the gelatin sponges makes it entirely possible that cells completely infiltrated the structures, especially by the 90 day culture period, and deposited their mineral matrix inside the structures; essentially within the recesses of the scaffold where it may be extremely difficult to get stain in or out consistently. Going forward, more accurate methods of measuring mineral matrix deposition will be utilized, however, for the preliminary nature of this study the ARS stain and its fairly positive results proved adequate in demonstrating the osteoinductive potential of the modified gelatin sponges tested here. 

Statistical analysis was also performed between the two graphs to determine if there were any significant differences in absorbance for a given scaffold on a particular time point. A total of six post cross-linked scaffolds and five +EDC scaffolds showed significant increases in absorbance values when compared to their cross-linked counterparts (Table 4). 

**Table 4:** Scaffolds with significant increases (*p* < 0.05) in absorbance values when comparing cross-linking methods.

	Post Cross-Linked	+EDC
Day 1 cells	+PRGF +HA	
Day 28	+PRGF	+PRGF +CW
	+PRGF +HA	
Day 28 cells		+CW
		+HA
Day 90	+PRGF	+PRGF +CW
	+PRGF +HA	+PRGF +HA +CW
Day 90 cells	+PRGF	

#### 2.3.3. DAPI Staining

**Figure 7:**
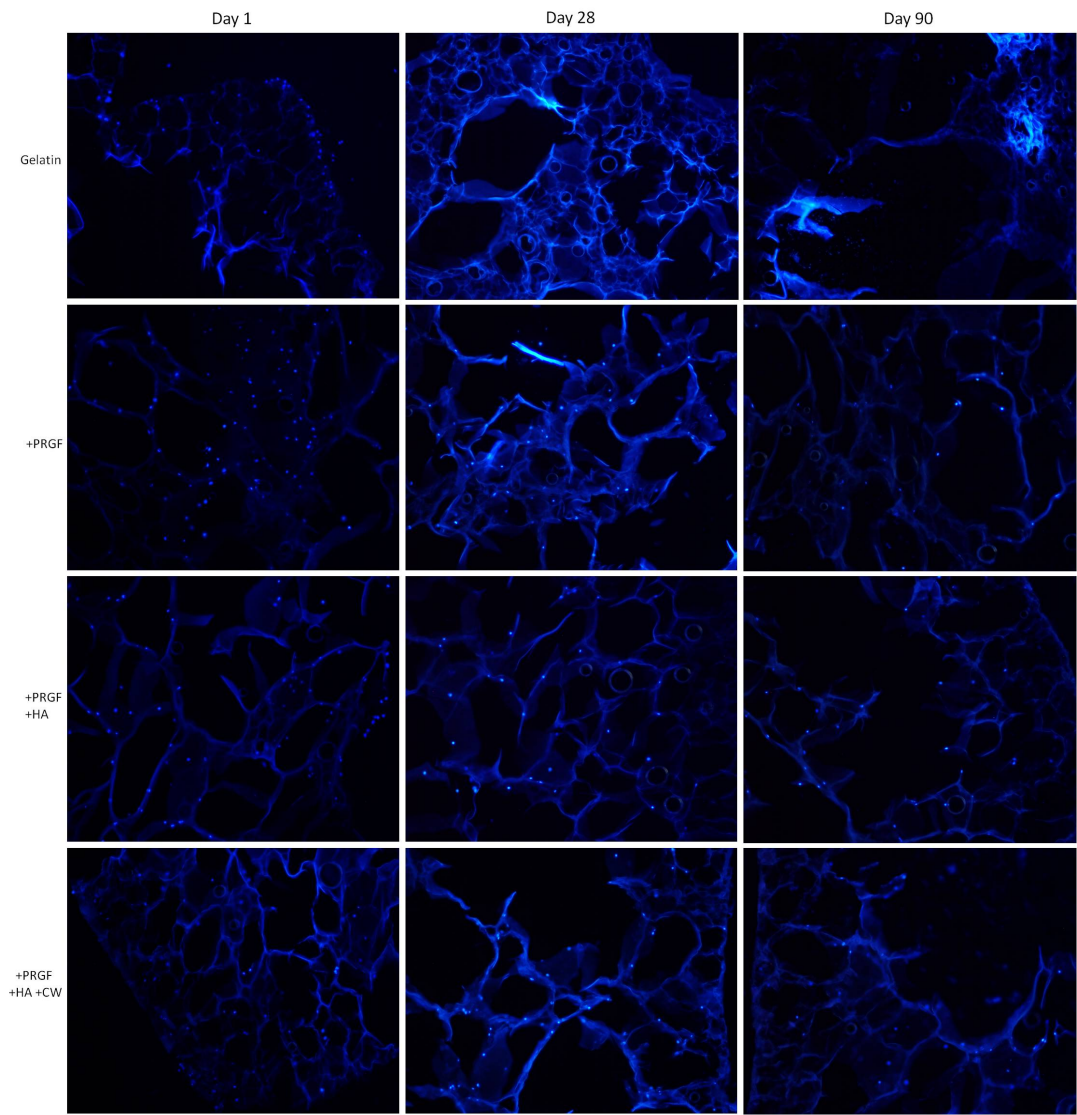
DAPI staining of post cross-linked scaffolds with MG-63 cells for 1, 28, and 90 days. Images taken at 20× magnification.

The SEM images provided an overview of how cells attached and produced matrix on the surface of gelatin composite sponges. Cell attachment and more importantly, infiltration into the scaffold, was analyzed via DAPI staining. Not all images are reported due to spatial constraints; however, the pure gelatin scaffolds (controls) are compared to three other scaffold compositions that facilitated a more pronounced infiltration of cells. In Figure 7, post cross-linked gelatin scaffolds are compared to +PRGF, +PRGF +HA, and +PRGF +HA +CW. After day 1, cells attached along the surface of the pure gelatin scaffold while the other scaffolds promoted cell infiltration and attachment (as evidence of blue cell nuclei visible throughout the cross section of the scaffold). With post cross-linked scaffolds, it appears that the addition of PRGF enhanced the attachment and infiltration of cells when compared to the gelatin control. The milieu of growth factors and cytokines contained within PRGF (some listed in Table 1) are known to be highly chemotactic as well as have the ability to induce cellular attachment, proliferation, and migration in a number of cell types. While the release of PRGF derived biomolecules was not specifically investigated in this study, it can be assumed that the growth factors and cytokines contained within the PRGF are being eluted from the scaffolds based upon previous studies conducted with the incorporation of PRGF into electrospun scaffolds [37,44]. These results suggest that these molecules remain active post-scaffold fabrication and ultimately increase the bioactivity of the scaffolds. 

**Figure 8:**
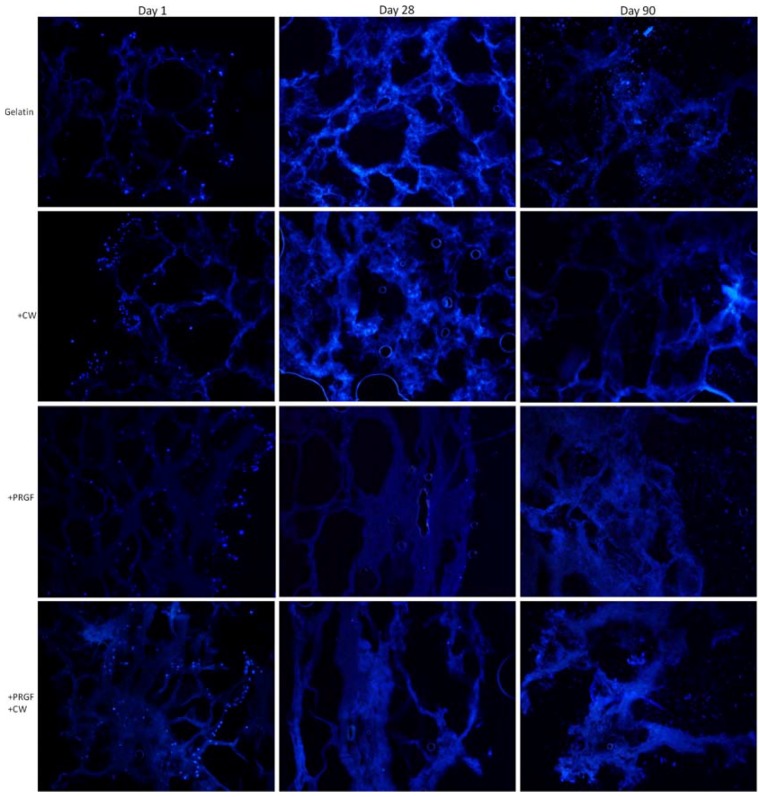
DAPI staining of +EDC cross-linked scaffolds with MG-63 cells for 1, 28, and 90 days. Images taken at 20× magnification.

Figure 8 compares the gelatin +EDC control to +CW, +PRGF, and +PRGF +CW +EDC. A similar trend was noticed with the +EDC scaffolds in that cells attached mainly to the control gelatin scaffold surface while the other scaffold compositions showed enhanced cell infiltration after day 1. However, it appears that more cells penetrated and attached to +EDC scaffolds when compared to post cross-linked scaffolds. By day 28 and 90, it became increasingly difficult to distinguish fluoresced cell nuclei on any scaffolds, independent of cross-linking methods. There appeared to be an abundance of newly created cell matrix at days 28 and 90, especially with +EDC scaffolds. This newly deposited matrix may have interfered with the staining, which commonly produced a uniform fluoresced scaffold making it difficult to discern individual cell nuclei and analyze day 28 and 90 DAPI images. With +EDC scaffolds, the addition of CW and/or PRGF appeared to promote cell attachment and penetration when compared to the gelatin +EDC control. 

### 2.4. Uniaxial Compression Testing

**Figure 9:**
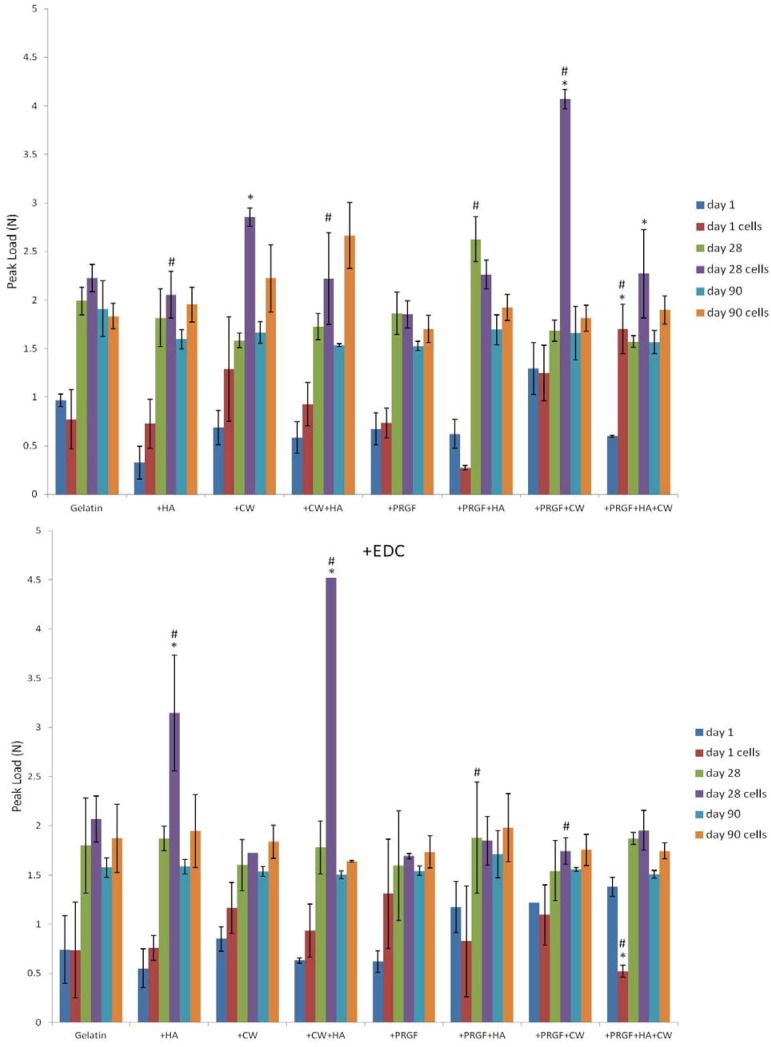
Peak load (N) of acellular and cellular scaffolds over 90 days incubation. * denotes a statistical difference (*p* < 0.05) between cells and no cells for a specific day and scaffold composition. # denotes a statistical difference (*p* < 0.05) when comparing cross-linking methods of each condition.

Peak load was recorded to determine the compressive strength of the cellular and acellular composite gelatin sponges incubated for 90 days (Figure 9). Each scaffold composition was compared to itself at each day to determine if the presence of cultured cells strengthened or weakened the scaffold over the duration of culture. Most of the post-gelation cross-linked scaffolds did not record a significant difference (*p* < 0.05) between cellularized and acellular scaffolds, meaning that the addition of cells did not significantly affect the scaffolds compressive strength positively or negatively. However, significant increases (*p* < 0.05) in peak load as a result of culturing cells were observed for +PRGF +HA +CW (day 1 and 28), +CW (day 28), and +PRGF +CW (day 28) post-gelation cross-linked scaffolds. Many of the scaffolds cross-linked during fabrication (+EDC) also did not record significant differences (*p* < 0.05) between cells and no cells when comparing scaffolds to themselves at a specific day. Significant increases (*p* < 0.05) in compressive strength were recorded for +HA (day 28) and +CW +HA (day 28) scaffolds suggesting the addition of cells improved scaffold strength. 

Upon comparing peak load between cross-linking methods, it was noticed that for the majority of the scaffolds, the method of cross-linking had no significant effect (*p* < 0.05) on the compressive strength for a specific scaffold on a particular day. Cross-linking during scaffold fabrication (+EDC) significantly increased (*p* < 0.05) the compressive strength of +HA (day 28 cells) and +CW +HA (day 28 cells) scaffolds. On the other hand, +EDC scaffolds showed significant decreases in peak load for +PRGF +HA (day 28 no cells), +PRGF +CW (day 28 cells), and +PRGF +HA +CW (day 1 cells) scaffolds.

## 3. Experimental Section

### 3.1. Fabrication of Gelatin Composite Sponges

All scaffolds were fabricated with a base solution of 30 mg/mL gelatin (Type B from Bovine skin, Sigma) in deionized (DI) water. For composite scaffolds, a total amount of 10 mg/mL of PRGF, HA, and/or CW were weighed, added to the 30 mg/mL gelatin solution, then sonicated if necessary (Table 5). Materials included HA nanopowder (particle size *<* 200 nm (BET), Sigma-Aldrich), CW (prepared by following a published protocol [22]), PRGF (created using published protocol [38]), and1-Ethyl-3-[3-dimethylaminopropyl]carbodiimide hydrochloride (EDC, Thermo Scientific). To create PRGF, fresh human whole blood from 3 donors was purchased, combined, and centrifuged (SmartPReP^®^ 2) to create PRP. PRP then underwent a freeze (−70 °C)–thaw (37 °C)–freeze (−70 °C) cycle to ensure platelet lysis and activation. Frozen PRP was then lyophilized to obtain a dry PRGF powder to be weighed and incorporated within the gelatin solution [38]. 

**Table 5:** Scaffold Components and Fabrication Concentrations.

	Amount (mg/mL) Added to Gelatin Solution	Sonicated
+HA	10	yes
+CW	10	yes
+HA+CW	5 (HA) and 5 (CW)	yes
+PRGF	10	no
+PRGF+HA	5 (PRGF) and 5 (HA)	yes (HA) then PRGF added
+PRGF+CW	5 (PRGF) and 5 (CW)	yes (CW) then PRGF added
+PRGF+HA+CW	3.33 (PRGF), 3.33 (HA), and 3.33 (CW)	yes (HA+CW) then PRGF added

4 mL of the prepared gelatin or gelatin composite solution was pipetted into a 35 × 10 mm Petri dish, refrigerated at 4 °C overnight to gel, and then slowly frozen at −15 °C overnight, −20 °C for 4 h, and −70 °C for 4 h. Frozen gel composites were lyophilized for 24 h then cross-linked for 18 h at room temperature in 50 mM EDC in ethanol [45,46,47,48]. To compare cross-linking methods, another set of solutions were made and 50 mM EDC was added directly to the composite solution before refrigerating to gel, the following steps remained the same as previously described (scaffolds denoted as +EDC). Using a Miltex biopsy punch, 6 mm discs were punched and used for all experiments.

### 3.2. Mass Loss

Two 6 mm scaffold punches were weighed as a unit for initial dry mass. Scaffolds were then disinfected (30 min ethanol followed by three 10 min washes of PBS) and transferred to a 48 well plate (two discs per well, n = 3). 500 µL of Dulbecco’s modified Eagle’s medium (DMEM) high glucose containing 10% fetal bovine serum (FBS) and 1% penicillin/streptomycin was added to each well. The two scaffolds per well were incubated in media at 37 °C and 5% CO_2_ with media changes every 7 days. Scaffolds were removed and weighed as a unit every 7 days up to 90 days. Hydrated scaffolds were massed and compared to original dry weights as a percentage to determine percent increase in scaffold mass. Scaffolds were air-dried post 90 day culture and compared to original dry weights to determine overall mass loss. 

### 3.3. Protein Release

To determine total protein content of each 6 mm scaffold disc, triplicates of one non cross-linked 6 mm discs of each scaffold type was immersed in 500 µL of 1× PBS. Uncross-linked scaffolds completely degraded within minutes at room temperature. Since scaffolds are primarily comprised of gelatin, the degraded byproducts are detectable using a general protein assay. Protein was quantified using a Pierce BCA Protein Assay (Thermo Scientific). Briefly, 25 µL of PBS containing the degraded scaffold contents was added to 200 µL of working reagent in a 96-well plate. The well plate was then incubated at 37 °C for 30 minutes, cooled to room temperature, and absorbance measured at 562 nm using a SpectraMax Plus 384 Microplate Spectrophotometer (Molecular Devices).

Scaffold release kinetics was studied by quantifying protein release from each scaffold over a period of 90 days. Triplicates of one 6 mm disc of each cross-linked scaffold type were incubated in 500 µL of 1× PBS at 37 °C with PBS replaced every 3 days. After 1, 4, 7, 10, 14, 21, 28, 56, and 90 days the PBS containing released scaffold contents in each well was analyzed for protein content using the Pierce BCA Protein Assay described above. 

### 3.4. Cell Attachment, Migration and Matrix Production

Triplicates of 6 mm discs of each scaffold composition were seeded with 50,000 osteoblast-like cells (MG-63 cells from a human osteosarcoma) and incubated at 37 °C and 5% CO_2_ in DMEM high glucose media containing 10% FBS and 1% penicillin/streptomycin with media changes every three days. After 1, 28, and 90 days, scaffolds were fixed in 10% formalin and stored at 4 °C until preparation for scanning electron microscopy (SEM) and fluorescent staining. For uniaxial compression testing, scaffolds were tested directly after being removed from the incubator.

#### 3.4.1. Scanning Electron Microscopy

Scaffolds were removed from formalin, briefly rinsed in PBS and water, and then subjected to ethanol dehydration (10 min soaks in 30, 50, 70, 90, and 100% ethanol, subsequently). Samples were air dried overnight, mounted on aluminum stubs, sputter coated in gold for 70 sec, and examined using a JEOL JSM-5610LV scanning electron microscope.

#### 3.4.2. DAPI Staining

Scaffolds were removed from formalin, immersed in a 30% sucrose solution in DI water for 48 hours at 4 °C to ensure displacement of all air bubbles, suspended in premium frozen section compound (VWR), and frozen at −70 °C overnight. 60 µm slices were cryosectioned using a Cryostat (Thermo) and transferred to microscope slides. Cryosectioned samples were then stained with 4'-6-diamidino-2-phenylindole (DAPI) stain for 5 min and imaged using a UV fluorescent microscope to display the location of cell nuclei. 

#### 3.4.3. Alizarin Red S Staining

Alizarin Red S (ARS) is a dye that selectively binds to calcium salts. ARS staining was used to quantify scaffold mineral content by modifying a published protocol [49]. ARS was performed on the 6 mm scaffold punches after 1, 28, and 90 days incubation in media with and without cells. After incubation scaffolds were stained with 40 mM alizarin red for 30 min then washed with DI water to remove any unbound stain. Scaffolds were then transferred to a 2 mL microcentrifuge tube containing 1.5 mL of 50% acetic acid to destain for 1 h at room temperature. 500 µL of the solubilized stain was added to 600 µL of 1 M NaOH to adjust the pH to 4.1. 200 µL of this solution was pipetted into a 96-well plate and absorbance read at 550 nm using a SpectraMax Plus 384 Microplate Spectrophotometer (Molecular Devices).

### 3.5. Uniaxial Compression Testing

Uniaxial compression testing was performed on acellular and cellularized 6 mm scaffold discs after 1, 28, and 90 days incubation in media. Mechanical testing was conducted by attaching an indenter (cylindrical, 2 mm diameter, plane-ended, stainless steel) to a MTS Bionix 200 Mechanical Testing System instrument with a 100 N load cell (MTS Systems Corp., USA). Indentation was performed perpendicular to the scaffold surface at the center of each scaffold disc. The discs were placed on a flat metal surface and kept hydrated with PBS. The indenter was lowered to the surface of the scaffolds and the following parameters were used: test speed of 0.5 mm/min, data acquisition rate of 10 Hz, a preload of 0.015 N, and a max indenter displacement of 90% of the scaffold thickness. Peak load was calculated by the MTS software TestWorks 4.0. Many scaffolds at later time points did not register a preload until later in the testing which resulted in the indenter moving through the entire scaffold until reaching the maximum load of 100 N when the intender contacted the metal plate. In these instances, the maximum peak load plateau, just before the maximum load was reached, was extracted from the graph and reported. 

### 3.6. Statistical Analysis

Statistical analysis was performed using JMP IN 9 statistical software (SAS Institute) to determine significant differences. Analysis of the data was based on a Kruskal-Wallis one-way analysis of variance on ranks and a Tukey-Kramer pairwise multiple comparison procedure. The results are presented in mean *±* standard deviation (SD). Unless otherwise specified all samples were run at a minimum of triplicate (n = 3) to ensure statistical significance.

## 4. Conclusions

In this study it was demonstrated that a lyophilized gelatin gel sponge, modified through the addition of PRGF, HA, and CW, demonstrated clear osteogenic potential when cultured with an MG-63 osteoblast-like cell line. These scaffolds, further modified through EDC cross-linking during gelation, were able to remain intact after 90 days in culture while exhibiting a controlled protein release. This tailorable rate of degradation is critical in a bone repair scaffold, where scaffold breakdown needs to match the ingrowth of new bony matrix to prevent catastrophic failure or the potential for micromotion or stress-shielding to occur. While this preliminary study failed to determine a clear optimal combination of gelatin and scaffold modifying agents (PRGF, HA, CW) to promote bone regeneration, it demonstrated that the use of a lyophilized gelatin gel sponge with the potential to absorb several times its weight in water was capable of eliciting cell infiltration into the structures as well as promoting the formation of cell-created mineral matrix. However, the +EDC scaffolds containing +PRGF+HA+CW performed well in all preliminary evaluations and need to be further investigated. Testing must be performed to more accurately determine the cellular interaction with these scaffolds, particularly the cellular response inside the structures. 
